# Effectiveness of long-term infliximab use and impact of treatment adherence on disease control in refractory, non-infectious pediatric uveitis

**DOI:** 10.1186/s12969-019-0383-9

**Published:** 2019-11-29

**Authors:** Virginia Miraldi Utz, Sabrina Bulas, Sarah Lopper, Matthew Fenchel, Ting Sa, Mitul Mehta, Daniel Ash, Daniel J. Lovell, Adam H. Kaufman

**Affiliations:** 1 Abrahamson Pediatric Eye Institute, Cincinnati Children’s Hospital Medical Center, Cincinnati, OH USA; 2 Department of Ophthalmology, University of Cincinnati, Cincinnati, OH USA; 3 Department of Biostatistics and Epidemiology, Cincinnati Children’s Hospital Medical Center, Cincinnati, OH USA; 4 Gavin Herbert Eye Institute, University of California, Irvine, CA USA; 5 Department of Medicine, University of Chicago, Chicago, IL USA; 6 Division of Rheumatology, Cincinnati Children’s Hospital Medical Center, Cincinnati, OH USA; 7 Cincinnati Eye Institute, Cincinnati, OH USA

**Keywords:** Uveitis, Iridocyclitis, Iritis, Infliximab, Biologic response modifier, Treatment adherence

## Abstract

**Background:**

Refractory non-infectious uveitis is a serious condition that leads to ocular complications and vision loss and requires effective systemic treatment to control disease. The effectiveness of long-term infliximab [IFX] in refractory non-infectious childhood uveitis and the impact of treatment adherence on disease control were evaluated.

**Methods:**

Retrospective, single-center study between December 2002 and April 2016 of 27 children with refractory non-infectious uveitis [17 with juvenile idiopathic arthritis, JIA] treated with long-term IFX [9+ months]. Disease activity was assessed prior to and while on IFX using the Standardization of Uveitis Nomenclature [SUN]. Number of visits per year with active uveitis was analyzed by repeated measures logistic regression analysis from 2 years prior to IFX initiation or from onset of uveitis until most recent visit on IFX. Incomplete treatment adherence was assessed for each visit and defined as any deviance in corticosteroid use, prescribed infusion frequency, and/or follow-up examination frequency.

**Results:**

Primary outcomes were sustained uveitic and systemic disease control prior to and during IFX treatment and the impact of incomplete adherence on uveitic disease control while on IFX. Secondary outcomes included corticosteroid and glaucoma medication requirement, ocular complications and need for surgical intervention. Mean age at IFX initiation was 10.4 ± 4.5 years; initial mean dose was 6.6 ± 2.2 mg/kg [and given at weeks 0, 2, 4 and q4 weeks thereafter for 93%]. Median duration on IFX was 35 [range 9–128] months. Prior to IFX, 14/27 patients had failed adalimumab ± methotrexate [MTX]; 21/27 failed MTX. IFX led to uveitis control in 89% and arthritis control in 76% (13/17). The odds ratio of having controlled disease after IFX was 4.1 (2.6, 6.4) compared to pre-treatment visits. Topical corticosteroids and glaucoma medications were statistically decreased (*p* = 0.007 right eye [OD], 0.003 left eye [OS] and *p* = 0.001 OD, *p* = 0.028 OS respectively). Incomplete adherence to treatment showed 10.3 times greater odds (7.1, 15.0) of having disease activity than full adherence.

**Conclusions:**

This study adds significantly to the IFX literature by documenting outstanding uveitis control with long-term IFX treatment in non-infectious pediatric uveitis patients. Higher dosage and shorter interval were utilized without adverse effects. Importantly, this is the first study, to our knowledge, to document the significant impact of treatment adherence on uveitis control.

## Background

Pediatric non-infectious uveitis [NIU] is an important cause of visual morbidity in children, with prevalence in the United States of 29/100,000 [1]. Ocular inflammation can be associated with systemic disease, most commonly Juvenile Idiopathic Arthritis [JIA] [1] or may be idiopathic. Vision loss from ocular complications such as band keratopathy, synechiae, cataracts, glaucoma, cystoid macular edema and hypotony occur in up to 75% of patients [2–5]. Visual acuity worse than 20/40 and 20/200 occurs in approximately 20 and 5% of patients respectively even with advances in therapeutic options [6]. Therefore, timely diagnosis and initiation of an effective management protocol are strongly indicated.

While local and systemic therapy with corticosteroids may be utilized as initial treatment, ocular [7, 8] and systemic [9] side effects of long-term administration warrant early implementation of corticosteroid-sparing therapy to improve visual outcomes [8, 10]. Most commonly, methotrexate is used as initial treatment in children, with response rate ranging from 50 to 75% of patients [6, 11]. For those who do not respond or incompletely respond, escalation to a biologic response modifier such as the tumor necrosis factor-alpha [TNF-α] inhibitors, adalimumab [ADA, Humira™] and infliximab [IFX, Remicade™], is appropriate to attempt to control disease activity [12–15].

Cytokines like TNF-α have been shown to play a role in the pathogenesis of uveitis, as evidenced by elevated TNF-α levels in the aqueous humor [16, 17]. IFX is a chimeric human/murine monoclonal antibody to TNF-α, that binds to free and membrane-bound forms and initiates a conformational change that inhibits binding to TNF receptors [18]. IFX has been used for pediatric uveitis for many years with variability in reported response rates ranging from 0 to 100% [19–26], with average response rate of 72% based on meta-analysis by Simonini and colleagues in 2014 [26].

To successfully manage and to treat uveitis, frequent appointments and complicated medication regimens are often needed. Adherence to both treatment and follow-up evaluations are paramount to treatment success of uveitis [27, 28]. Children represent a vulnerable population, and treatment success may largely depend on the partnership between the caregivers and the clinicians to promote adherence. Uveitis with or without JIA has the potential to leave residual and permanent disability, which has life-long consequences for these children. One of the benefits of IFX is that the clinician can track infusion administration. Therefore, understanding the impact of adherence on treatment efficacy and disease control is an important endeavor. In this study, we evaluate the long-term use of IFX to sustain control of inflammation and impact of treatment adherence in a single center study of children with recalcitrant NIU. The number of patients in this study is larger than relevant publications in the literature.

## Methods

Institutional review board approval was obtained, and the study adhered to the Declaration of Helsinki. The ophthalmology and rheumatology medical records of 43 children with pediatric non-infectious uveitis from December 30, 2002 and April 30, 2016 at Cincinnati Children’s Hospital Medical Center [CCHMC] or Cincinnati Eye Institute [CEI] were reviewed. Potential patients were identified by ICD-9 and ICD-10 billing codes. Patients were included in the study if they had received IFX treatment for a minimum of 9 months, had complete ophthalmology and rheumatology records available spanning ≥2 years prior to IFX treatment initiation or from uveitis onset to the start of IFX treatment if less than 2 years. Complete ophthalmology records included all visit notes and examination findings and were collected systematically via an IRB-approved data collection sheet for each patient.

### Data collection

Information on all patients with non-infectious uveitis treated with IFX was collected and managed in REDCap [Research Electronic Data Capture] [29]. Data included demographic information, ophthalmologic and rheumatologic examinations, results of laboratory testing, systemic diagnoses, all prior and current systemic treatments, IFX dosage and frequency, treatment adherence, ocular medications [dose and route of administration] prior to and during treatment with IFX (Additional file 1: Table S1). The anatomic location and grading of intraocular inflammation were in accordance with the Standardization of Uveitis Nomenclature [SUN] Classification Scale [30]. JIA was diagnosed by a pediatric rheumatologist based on the International League of Associations for Rheumatology [ILAR] classification criteria [31]. Failure of prior systemic steroid-sparing therapy was defined as persistent or worsening uveitis and/or failure to wean ocular corticosteroids to ≤2 drops of prednisolone acetate 1% [PA] daily and/or failure to control systemic disease. Incomplete adherence was defined at each visit while on infliximab as 1) missed appointment without rescheduling within 2 weeks, 2) missed infusion or rescheduled infusion outside the prescribed dosing interval, and/or 3) self-reported incomplete adherence to prescribed systemic or topical medication. Non-adherence to follow-up appointments was assessed by reviewing the follow-up recommended in the ophthalmologist’s last disposition relative to the next appointment visit date. Non-adherence to infusion was determined reviewing the dates of each infusion procedure within the electronic medical record. The use of systemic medications such as methotrexate and topical corticosteroids was self-reported. If the self-reported treatment did not correspond to the prescribed frequency (based on the last visit note) or patient/ family admitted to missing dosages, this was considered incomplete adherence.

### Outcome measures

The clinical examination prior to starting IFX was compared to the most recent visit while on IFX and included best-corrected visual acuity [BCVA], intraocular pressure [IOP], ocular inflammation [30], and presence or absence of active arthritis when applicable. Ocular complications and surgical interventions prior to and while on IFX were assessed. Complications included band keratopathy [BK], ocular hypertension [OHT], glaucoma, anterior and posterior synechiae, hypotony, and cystoid macular edema [CME]. Complications were as defined by Woreta et al. [5], with the exception of ocular hypertension and glaucoma in which standard pediatric-specific criteria were utilized [32]. The percent with active arthritis and/or uveitis, weekly topical corticosteroid burden, and glaucoma medication burden were evaluated. For those on difluprednate, the dosages were converted to prednisolone acetate 1% [PA] equivalent [2 drops PA = 1 drop difluprednate] [33]. We also calculated the percentage reaching ≤2 drops of PA [7] as well as steroid-free remission defined as no systemic or local corticosteroids for the duration of the follow-up period [34]. IFX-related adverse events were evaluated.

In addition to pre- and most recent visit comparisons, we sought to analyze longitudinal disease activity and complications by documenting the following in each eye for all visits 2 years prior to [or from the onset of uveitis if < 2 years] until the most recent visit on IFX: uveitic disease activity; corticosteroid use [route/dose/frequency]; and glaucoma medication use. Adherence was evaluated for each visit while on IFX. Active anterior segment inflammation corresponded to cell grade 0.5+ or higher or vitreous haze > 1+ as described by SUN and/or described by terms such as “active, worsening inflammation, or disease progression.” [30, 34] Controlled uveitis corresponded to findings of < 0.5+ cell or descriptive terms such as “quiet, quiescent, and no active inflammation.” [34] Controlled uveitis was further subdivided into those who were controlled on ≤2 drops of PA/day versus those requiring > 2 drops PA/day.

Best-corrected visual acuity [BCVA] was converted to logMAR [the logarithm of the minimal angle of resolution or decimal-form of BCVA] was compared prior to and at the most recent visit on IFX by a paired t-test. Visits with active disease per year before and during IFX treatment were compared via a repeated measures logistic regression analysis. The effect of adherence on disease control for each clinical visit while on IFX was calculated by a repeated measures logistic regression analysis. Baseline and at most recent visit weekly topical corticosteroid burden before and while on IFX were compared using a paired t-test. We assessed topical glaucoma medication use via Wilcoxon signed-rank analysis at baseline, defined as before or within six months of IFX initiation, and during IFX use starting six months into treatment. The six-month timeframe was selected to allow adequate time for full effectiveness of IFX in combination with being able to slowly taper topical steroids and subsequently taper glaucoma medications accordingly. We utilized a Fischer’s exact test to evaluate the significance of complications pre- and during infliximab. SAS 9.4 M4 software was used for all analyses [SAS Institute Inc., Cary, NC.] Significance for all tests was set a priori at α = 0.05.

## Results

### Demographic and baseline characteristics

A total of 27 [18 female] patients’ records were included with a median follow-up time on IFX was 35 months [range 9–128]. Sixteen patients were excluded secondary to IFX treatment duration < 9 months [*n* = 9] or incomplete records [*n* = 7]. Demographic and baseline clinical and disease characteristics of the patients are presented in Table 1. Importantly, 52% [14] of patients had failed standard weight-based dosing of ADA therapy either with or without concomitant MTX for either joint or ocular disease, and three of the patients had failed weekly weight-based ADA treatment. Patients on average had 15 ± 8 [median 13] visits prior to IFX start date and 22 ± 15 [median 16.5] visits while on IFX. Thirteen patients had ≥2 years of follow-up prior to IFX initiation, and 21 patients had ≥1 year of follow-up prior to IFX start. Those with < 2 years of follow-up had onset of disease at or within the 2 years prior to IFX start.

**Table 1:** Demographics and Baseline Characteristics of Pediatric Patients Treated with Infliximab

Demographics and Clinical Characteristics	Number of patients (% of population)
Female, *n* = 27	18 (67%)
Race, n = 27	
Caucasian	22 (82%)
African-American	2 (7%)
Hispanic	2 (7%)
Asian	1 (4%)
Juvenile idiopathic arthritis, *n* = 17	17 (63%)
Extended Oligoarticular	3 (3 ANA+)
RF-negative, Polyarticular	11 (9 ANA+)
Enthesitis-related	1 (HLA-B27 +)
Juvenile psoriatic arthritis	1 (ANA+)
Undifferentiated	1 (ANA+)
Idiopathic	7 (26%)
Sarcoidosis	1 (4%)
HLA-B27-associated	1 (4%)
Other	1 (4%)
Serology, n = 27	
ANA positive	14 (52%)
RF positive	0
HLA-B27	1 (4%)
HLA-B51^a^	1 (4%)
Uveitis Laterality, n = 27	
Bilateral	23 (85%)
Unilateral	4 (15%)
Uveitis Location, n = 27	
Anterior	19 (70%)
Intermediate	5 (19%)
Anterior Intermediate	2 (7%)
Panuveitis	1 (4%)
Age (Mos) of JIA Diagnosis (n = 17) Ave ± SD (median)	43.6 ± 36.0 (23)
Age (Mos) of Uveitis Diagnosis (n = 27) Ave ± SD (median)	86.5 ± 53.5 (76)
Duration of uveitis (Mos) prior to IFX start date, (*n* = 27) Ave ± SD (median)	39.6 ± 36.0 (22)
Follow-up Duration (Mos) on IFX, (n = 27) Ave ± SD (median)	41.6 ± 31.2 (35)
Previous Treatments prior to IFX Start^b^, n = 27	
Methotrexate (MTX)	21 (78%)
Adalimumab (ADA)^c^	14 (52%)
Etanercept (ETN)	1 (4%)
Oral prednisone within 2 years prior to IFX start	7 (65%)
Indications for IFX	
Active uveitis in ≥1 eye pre-IFX [n = 27]	19 (70%)
Active uveitis OR < 0.5+ cell, but > 2 drops PA 1% to control disease [n = 27]	24 (90%)
Active JIA (joint disease) [n = 17]	11 (65%)

^a^Not routinely tested

^b^No patients were treated with cyclosporine or mycophenolate

^c^Adalimumab was dosed at 20 mg every other week for patients 15 kg to < 30 kg and 40 mg for patients ≥30 kg. Three patients failed weekly weight-based therapy of adalimumab

Abbreviations: *Mos* months, *Ave* average, *SD* standard deviation, *PA 1%* Prednisolone acetate 1%, *MTX* methotrexate, *ADA* adalimumab, *IFX* infliximab, *RF* rheumatoid factor, *ANA+* anti-nuclear antibody positivity

Mean initial dosing of IFX was 6.6 ± 2.2 mg/kg [median = 6.2 mg/kg] dosed every 2 weeks for the first 4 weeks, and then every 4 weeks thereafter for 92.6% [*n* = 25] of the population. Dosing was increased in 6 [22.2%], decreased in 10 [37.1%], and maintained in 11 [40.7%] over the follow-up period based on treatment response. Ten patients [37%] were on 10 mg/kg or more at some point in the treatment interval. No biosimilars were utilized. Twenty-six [96.3%] patients received concurrent MTX treatment [0.5 ± 0.3 mg/kg [5–25 mg] once weekly, 65% subcutaneous and 35% oral route] with IFX for at least a portion of the treatment interval; 78% were on MTX before starting IFX and 19% started MTX after beginning IFX treatment to prevent the development of human anti-chimeric antibodies [HACAs]. One patient was on concurrent treatment with leflunomide.

At the baseline slit lamp examination, 70% of patients had active uveitis in at least one eye, with 59% [10/17] of patients with active disease having an associated diagnosis of JIA. Of the 30% patients who were documented “controlled” at baseline, 62.5% [5/8] were on > 2 drops of PA/day. Active joint disease was the indication for IFX for remaining three patients.

### Disease activity

At the most recent visit, 52% (14/27) of patients had no uveitis activity without topical steroids, and 89% (24/27) of patients were controlled with ≤2 drops PA/day. Proportion of patients with controlled uveitis increased from 30% before IFX to 89% on IFX. Joint disease in the JIA patients was controlled in 76%. Of those who failed IFX for either uveitis, arthritis or both, common features are a systemic association and development of HACAs in three (Additional file 2: Table S2) Results from the logistic regression demonstrated that patients were 4.1 times more likely to have active ocular disease in one or more eyes at any clinical visit before starting IFX compared to while on IFX treatment [*p* < 0.001, CI: 2.6, 6.4]. Sub-group analysis of the 17 patients with JIA did not yield statistically significant predictive results [*p* = 0.18, CI 0.868, 2.099]. Patients who met the criteria for incomplete adherence while on IFX were 10.3 times as likely to have active disease at any visit compared to those who were adherent to treatment and follow-up [*p* < 0.0001, CI: 7.1, 15.0]. Ten patients adhered to all treatments and visits, while 17 patients had evidence of incomplete adherence identified at one or more visits.

### Corticosteroid burden

At the baseline examination, 63% patients were on > 2 drops of PA/eye/day. After receiving IFX treatment for a mean of 41.6 ± 31.2 months [median 35 months, range 9–128 months], 89% of patients met the topical steroid threshold of ≤2 drops PA/eye/day in both eyes and 59% of patients had discontinued use of all topical drops at the most recent visit and were in steroid-free remission. The paired t-test showed a significant reduction in weekly PA equivalent dosage equivalent [*p* = 0.007 OD, *p* = 0.003 OS]. Seven patients required oral prednisone within the 2 years prior to starting IFX. Two patients received oral prednisone while on IFX: one for active joint disease and the other for pars planitis. Seven patients required periocular steroids within the interval 2 years prior to or from onset of uveitis to start date of IFX. Two patients received periocular steroids while on IFX: one at the time of cataract surgery for post-surgical inflammatory control and the second for retinal vasculitis.

### Ocular complications

Mean visual acuity at baseline [OD: 0.2 ± 0.4, 20/32; OS: 0.1 ± 0.1, 20/24] and at the most recent visit [OD: 0.20 ± 0.5, 20/32; OS: 0.1 ± 0.2, 20/24] was not statistically different [*p* = 0.09 OD, *p* = 0.10 OS]. Complications at baseline were present in a majority of patients (Table 2). Cataracts developed in five patients [3 eyes OD, 5 eyes OS] while on IFX, but none of these eyes required surgery over the follow-up (see Additional file 3: Table S3). Cataract surgery was required in two patients [7.4%] [3 eyes] while on IFX, but these cataracts were present prior to IFX initiation in both patients and need for inflammatory control prior to cataract surgery was the rationale for beginning IFX for one patient. There were no episodes of CME in any patient while on IFX.

**Table 2:** Ocular Complications Pre and During IFX Treatment Follow-up, n = 27

	Pre-IFX	During IFX	*P*-value
Band keratopathy	3	0	0.24
Cystoid Macular Edema	5	0	0.051
New cataract diagnosis	13	5	0.52
New glaucoma diagnosis	7	0	**0.02**
New glaucoma suspect diagnosis	11	0	**0.003**
Cataract Surgery	3	2^a^	1.00
Glaucoma Surgery	5	2^b^	0.42

^a^Cataracts that required surgery while on IFX were present prior to IFX initiation. In both patients, absolute control of inflammation off topical steroids prior to cataract surgery was rationale for the decision to start IFX

^b^Glaucoma surgery was performed in one of these patients within a month of starting IFX

Seven patients [26%, 14 eyes] had a diagnosis of glaucoma and 11 patients [41%; 21 eyes] had a diagnosis of glaucoma suspect based on the presence of ocular hypertension. No patients developed glaucoma or had ocular hypertension while on IFX, and this was statistically significant [*p* = 0.02, *p* = 0.003 respectively] (Table 2). Glaucoma surgery was performed on two patients with pre-existing glaucoma shortly after being placed on IFX [< 3 months]. Lastly, topical glaucoma medication burden was significantly reduced while on IFX [*p* = 0.0013 OD and *p* = 0.0278 OS].

### Complications of IFX treatment

In our population, IFX was well-tolerated, with no cases of severe adverse events or opportunistic infections over the follow-up period, even in those on higher dosages. Six patients [22.2%] experienced side effects of IFX treatment, four of whom developed HACAs. In two of the four patients who developed HACAs, uveitic disease and/or joint disease became uncontrolled, leading to discontinuation of IFX. One patient had a hypotensive episode with transfusion leading to discontinuation, although this patient’s ocular disease was controlled at the most recent visit. The four patients who developed HACAs were treated subsequently with the following: patient 1: abatacept followed by tocilizumab; patient 2: ADA 40 mg weekly (ADA naïve); patient 3: ADA weekly, followed by abatacept and subsequently tocilizumab; and the fourth patient was lost to follow-up. The remaining two experienced headaches during an infusion but did not discontinue IFX. Demographics and clinical characteristics of those who failed infliximab treatment can be found in Additional file 2: Table S2.

## Discussion

IFX has a long track record of efficacy for treating pediatric uveitis [19–26], however, several key findings can be learned from this study: [1] IFX was efficacious for long-term control of disease in the majority of this population and represented a viable alternative for those who had failed treatment with ADA for either uveitis and/or joint disease; [2] Topical corticosteroid and glaucoma medication was reduced on IFX; [3] Treatment adherence is key to achieving optimal effectiveness.

Firstly, we found a high level of IFX effectiveness in our patient population. Twelve of the 14 patients who failed ADA treatment prior to IFX had active uveitis or required > 2 drops of PA/day to control disease. After changing to IFX, all but one patient was controlled at their most recent visit and all had a steroid regimen of ≤2 drops of PA/day with half of the patients discontinuing ocular steroids entirely. This study did not directly compare efficacy of ADA to IFX; however, this study supports previous studies that changing TNF-alpha inhibitors represents a viable option for those failing ADA as initial treatment [35].

Significant variability of IFX response rate for pediatric uveitis is present in the literature ranging from 0 to 100% [19–26, 36–39], with average of response rate of 72% based on a meta-analysis by Simonini and colleagues in 2014 [26]. Historically, the frequency and dosage has been variable for uveitis extrapolating from the treatment of other autoimmune disorders such as rheumatoid arthritis and inflammatory bowel disease, in which dosages of 3–5 mg/kg dosed every 6 to 12 weeks are adequate to control disease [40–43]. Several pediatric uveitis studies that report less favorable results and/or loss of effectiveness with IFX dosed at 5 mg/kg dose every 6–8 weeks after initial q2week loading doses [20, 36–38]. Previous literature suggests that higher dosages of IFX at an infusion frequency of every 4 weeks may be needed to effectively control pediatric uveitis [22, 39].

Higher concentrations may allow more adequate penetration of the ocular compartment as compared to joint or gastrointestinal system. The pharmacokinetic and pharmacodynamic aspects of biologic response modifiers are complex and individualized optimal dosing predictions have only been determined for inflammatory bowel disease [44, 45]. For non-infectious pediatric uveitis, optimal dosing of IFX and treatment interval has been determined empirically. A well-designed prospective trial may help to guide patient-specific dosing for non-infectious uveitis based on pharmacokinetic and pharmacodynamic parameters.

Importantly, no severe adverse events occurred in the present study. The frequency of HACAs in our population [15%] was less than that observed in prospective pediatric cohorts with JIA with or without uveitis [23–37%] reported previously [46, 47]. The lower frequency observed is likely an underestimate as HACAs were measured only in those failing therapy. Alternatively, higher and more frequent dosing used to treat uveitis may be protective against antibody formation as demonstrated by Aeschilmann and colleagues [46]. The routine use of methotrexate or other DMARDs while on an IFX may also prevent antibody formation. Randomized protocols combined with routine serum IFX levels and HACAs are needed to delineate the role of dosage and frequency of IFX and use of concomitant DMARDs in preventing HACA formation.

Secondly, there were relatively few ocular complications and surgical interventions in our patients during IFX treatment: no patients developed band keratopathy, CME or new diagnoses of glaucoma suspect or glaucoma while on IFX. The two patients’ cataracts that required surgery while on IFX were already present prior to the initiation of IFX, and IFX was utilized to control disease prior to the indicated surgery. Likewise, one of the patients who required glaucoma surgery while on IFX had surgery within 3 months of starting IFX. The high rate of inflammatory control and low incidence of complications provides further support for IFX effectiveness at this dosing and frequency.

Coinciding with decreased incidence of ocular complications, IFX treatment minimized known detrimental complications associated with prolonged steroid usage, such as OHT, glaucoma, and cataract development [3–6, 9]. Although some patients were “quiet” and/or “controlled” at baseline, the high steroid burden required to control disease represented an insufficient long-term treatment plan, as patients on chronically high doses of topical corticosteroids are more likely to suffer complications [7, 48]. In order to capture the effectiveness of IFX in patients who had quiet disease at baseline due to aggressive topical and systemic treatment regimens, we utilized steroid burden [drug, potency, dose, and frequency] as a measurement marker. All of the patients who were controlled at the most recent visit on IFX were on ≤2 drops of PA, and a majority were in steroid-free remission. Interestingly, glaucoma medication paralleled the reduction in corticosteroids, likely reflecting resolution of ocular hypertension associated with reduced chronic corticosteroid use [48]. The reduction in topical corticosteroid therapy further supports long-term control on IFX compared to baseline.

Finally, this is the first study to our knowledge that assesses the impact of treatment adherence on disease activity in children with uveitis. Patients had a 10.4-fold increased risk of having active disease with incomplete adherence documented at any given visit while on IFX. Treatment adherence as defined by the World Health Organization is a “patient-centered term” describing “the extent to which a person’s behavior – taking medication, following a diet, and/or executing lifestyle, corresponds with the agreed recommendations from a health care provider” [49]. Treatment adherence has been extensively studied in the adult population, most notably for patients treated for primary open angle glaucoma [50–53]. For example, in the adult glaucoma literature, simplified dosing regimens and/or decreased frequency of dosing has been shown to improve reports of adherence [54, 55]. Likewise, recommendations have been proposed to increase adherence in the adult population with uveitis [28]. However, risk factors and interventions for incomplete treatment adherence have not been rigorously studied in children with uveitis.

Children represent a unique population: parents or guardians schedule and transport children to appointments and/or infusions, and treatments often depend on parents to administer medications at home in most cases. Adherence may be even more difficult to discern in older children or teenagers who self-administer drops or treatment, where adherence is a shared responsibility. The asymptomatic nature of JIA-like chronic iridocyclitis in which inflammation may be indolent and effects of damage may not be appreciated until the disease advances further, complicates treatment adherence. Therefore, further research is clearly needed to determine the risk factors for incomplete treatment adherence and barriers to adherence in children with uveitis. To address incomplete adherence, a multi-disciplinary approach that includes a pediatric rheumatologist and ophthalmologist combined with a social worker, devoted nurse and/or behavioral psychologist may prove beneficial. Some considerations include efforts in education of the disease process and consequences, reducing appointment burden by consolidating appointments/infusions, simplifying treatment protocols and uniform instructions and/or charts to document medication administration. Further research in this area will help to define risk factors and implement effective strategies to improve adherence.

Strengths of this study include patient numbers and longitudinal follow-up at a single center for biologic medication management and evaluation of adherence on treatment effectiveness. Because of previous reports of initial control on IFX with waning effectiveness with long-term follow-up [36–38], we selected patients who had long-term follow-up of nine months or more. The long mean duration of follow-up is another strength of this study. A maintained response was observed in a vast majority of patients over the treatment interval. We did not include patients with short-term follow-up, as previous studies had demonstrated no difference between six months on IFX and one year.

Limitations of this study include its retrospective nature, heterogeneity of disease, and lack of a control group. As a practice pattern at CCHMC, patients with more severe disease tend to be placed on IFX as an initial TNF-alpha inhibitor since the high dose protocol yields rapid control and confirmed adherence. Thus, the successful outcomes in this study may actually be more significant due to this practice. Additionally, success with adherence may also be more significant in this group of patients since the highly adherent regimen of IFX infusions was used in patients that may have been selected due to higher risk of poor adherence. One of the other limitations to our statistical model was the variable appointment interval, which was based on disease activity. Therefore, a patient may have more visits a specific year compared to prior or future years. Likewise, in terms of incomplete treatment adherence, missed IFX infusions and doses of concurrent systemic therapy [i.e. MTX] have delayed and/or variable effects of an unknown duration on disease activity, and therefore the effects of interval incomplete adherence for any particular office visit may not by readily discernable for that specific visit. For example, a patient may miss an IFX infusion and be quiet on next follow-up, but then develop activity 2–3 months later after missing several doses. While side effects of IFX treatment were minimal, long-term toxicity [10–15 years] cannot be determined over the follow-up period. Larger, prospective trials are needed to confirm and expand on these findings.

## Conclusions

In conclusion, IFX is remarkably safe and effective for long-term treatment of non-infectious pediatric uveitis. Higher dosage and shorter intervals may be necessary to achieve successful control in a greater percentage of patients. Corticosteroid burden, glaucoma medication burden, and development of complications were reduced by IFX treatment. Incomplete adherence greatly increased the odds of having active disease while on IFX and further quality improvement measures need to be studied and implemented to improve adherence in this vulnerable population.

## Supplementary information

**Additional file 1: Table S1.** Standard Collection Form for Study Patients.

**Additional file 2: Table S2.** Demographic, Clinical and Treatment History of Non-Responders (or Loss of Initial Response) to Infliximab Therapy. The demographic, clinical and treatment history of patients who failed IFX for arthritis or uveitis are listed. As stated in the text, no major trends were noted, however, a high proportion had associated systemic disease. JIA = Juvenile idiopathic arthritis; ANA + = anti-nuclear antibody positive; RF- = Rheumatoid factor negative; PA = polyarticular; OU = both eyes, mos = months; OD = right eye; OS = left eye; IFX = infliximab; PA = prednisolone acetate 1%; MTX = methotrexate; ADA = adalimumab; N/A = not applicable; HACAs = human antichimeric antibodies.

**Additional file 3: Table S3.** Ocular Complications Per Eye Pre and During Infliximab treatment (Per Eye, Right eye *n* = 25, Left eye n = 25). Ocular complications present before and while on IFX are listed by eye (as opposed to by patient) involved. We did statistical analysis on the complications per patient, please refer to Table 2. Abbreviations: OD – right eye, OS – left eye. *Cataracts that required surgery while on IFX were present prior to IFX initiation. In both patients, absolute control of inflammation off topical steroids prior to cataract surgery was rationale for the decision to start IFX. ** Glaucoma surgery was performed in one of these patients within a month of starting IFX.

## Data Availability

The datasets used and/or analyzed during the current study are available from the corresponding author on reasonable request.

## Abbreviations

ADA: Adalimumab
BCVA: Best corrected visual acuity
BK: Band keratopathy
CCHMC: Cincinnati Children’s Hospital and Medical Center
CEI: Cincinnati Eye Institute
CI: Confidence interval
CME: Cystoid macular edema
HACAs: Human anti-chimeric antibodies
IFX: Infliximab
ILAR: International League of Associations for Rheumatology
IOP: Intraocular pressure
JIA: Juvenile idiopathic arthritis
MTX: Methotrexate
NIU: Non-infectious uveitis
OD: Right eye
OHT: Ocular hypertension
OS: Left eye
PA: Prednisolone acetate 1%
SUN: Standardization of Uveitis Nomenclature
TNF-α: tumor necrosis factor-alpha
