# Supplementary material for: Effectiveness of long-term infliximab use and impact of treatment adherence on disease control in refractory, non-infectious pediatric uveitis
Source: Pediatr Rheumatol Online J. 2019 Nov 29;17:79. doi: 10.1186/s12969-019-0383-9 (PMC6884783; doi:10.1186/s12969-019-0383-9)
Supplement: Supplementary file 1 — Additional file 1: Table S1. Standard Collection Form for Study Patients. [file 12969_2019_383_MOESM1_ESM.docx]

Additional file 1: Table S1. Standard Data Collection Form

| Study ID Number | | DOB | | | | | Gender | Race | | | |  |  |  |
| --- | --- | --- | --- | --- | --- | --- | --- | --- | --- | --- | --- | --- | --- | --- |
|  |  |  |  |  |  |  |  |  |  |  |  |  |  |  |
|  |  |  |  |  |  |  |  |  |  |  |  |  |  |  |
| Diagnosis (JIA or other):  **Specify subtype of JIA (ILAR)*  **Please specify other: e.g. sarcoidosis, idiopathic, etc)* | | Age at JIA (systemic) diagnosis (mo) | | | Age of uveitis diagnosis (mo) | | | | | Serology: +(positive), -(negative), n/a | | | | |
|  |  |  |  |  |  |  |  |  |  | ANA+/-: | | | | |
|  |  |  |  |  |  |  |  |  |  | RF+/- | | | | |
|  |  |  |  |  |  |  |  |  |  | HLA-B27+/- | | | | |
| Dates (beginning-end) of active uveitis episodes (Specify OD/OS/OU)  Specify incoming & outgoing topical drugs+ dosage | | | | | | | | | | | | | | |
| Ocular Surgical Procedures (type, date): | | | | | | | | | | | | | | |
| Systemic meds dosage history (drug, mg/kg, frequency, route); Reason for discontinuation | | | | | | | | | | | | | | |
| Adalimumab (Humira)  dosage & frequency history | | Infliximab (Remicade)  dosage & frequency history | | | | | | | | Other BRM dosage & frequency history | | | | |
| Adverse Effects from Medications (med d/c, description) | | | | | | | Noncompliance over the course of follow-up (type, dates, date(s) of worsening disease if applicable, cell grade/number, OD/OS/OU) | | | | | | | |
| Initial eye exam (date):  ADA or IFX or Other | | | | | | | Last/most recent exam (date):  ADA or IFX or Other | | | | | | | |
| BCVA *(Snellen L/B/S; HOTV L/B/S; LEA L/B/S; Allen L/S)* | | | | | | | BCVA *(Snellen L/B/S; HOTV L/B/S; LEA L/B/S; Allen L/S)* | | | | | | | |
| OD: | | OS: | | | | | OD: | | | | OS: | | | |
| Cell | | Flare | | | | | Cell | | | | Flare | | | |
| OD: | OS: | OD: | | OS: | | | OD: | | OS: | | OD: | | | OS: |
| KP(+/-) | | IOP (mm Hg) | | | | | KP(+/-) | | | | IOP (mm Hg) | | | |
| OD: | OS: | OD: | | OS: | | | OD: | | OS: | | OD: | | | OS: |
| Cataract (+/-, type) | | Synechiae (+/-) | | | | | Cataract (+/-, type) | | | | Synechiae (+/-) | | | |
| OD: | OS: | OD: | | OS: | | | OD: | | OS: | | OD: | | | OS: |
| Vitreous cell (+/-, grade) | | C/D ratio | | | | | Vitreous cell (+/-, grade) | | | | C/D ratio | | | |
| OD: | OS: | OD: | | OS: | | | OD: | | OS: | | OD: | | | OS: |
| Drops: | | | | | | | Drops: | | | | | | | |
| JIA/systemic dx = active, controlled, remission | | | | | | | JIA/systemic dx = active, controlled, remission | | | | | | | |
| Iritis OD= active, controlled, remission  Iritis OS= active, controlled, remission | | | | | | | Iritis OD= active, controlled, remission  Iritis OS= active, controlled, remission | | | | | | | |
| **Other Complications Over Course of Follow-up** | | | | | | | | | | | | | | |
| Glaucoma suspect/diagnosis (Y/N)  OD/OS/OU  IOPmax+ date (OD/OS):  Topical meds for IOP: (Y/N)  List All:  Pachymetry  SD-OCT:  VF: | | | | | | | Cystoid Macular Edema (Y/N)  OD/OS/OU  SD-OCT:  Treatment required:  Band Keratopathy (Y/N)  OD/OS/OU  Treatment required: | | | | | | | |
| **Topical Drops Regimen History** | | | | | | | | | | | | | | |
| OD | | | | | | OS | | | | | | | | |
| Reached level of < 3 drops PF per day?  ( **Y** / **N** / **n/a** ) *if yes, date*:  *drug/dosage*: | | | | | | Reached level of < 3 drops PF per day?  ( **Y** / **N** / **n/a** ) *if yes, date*:  *drug/dosage*: | | | | | | | | |
| Tapered from PF to FML/lower strength steroid?  ( **Y** / **N** / **n/a** ) *if yes, date*:  *drug/dosage*: | | | | | | Tapered from PF to FML/lower strength steroid?  ( **Y** / **N** / **n/a** ) *if yes, date*:  *drug/dosage*: | | | | | | | | |
| Reached discontinuation of all drops?  ( **Y** / **N** / **n/a** ) *if yes, date*:  *drug d/c*: | | | | | | Reached discontinuation of all drops?  ( **Y** / **N** / **n/a** ) *if yes, date*:  *drug discontinued*:  *Duration of time off all drops*  *(disease free):* | | | | | | | | |
| Eye disease: < 3 drops PF per day (Y/N) and quiet disease (Y/N) on systemic treatment  for 2 or more years? | | | | | | | | | | | | | | |
| Biologic treatment discontinued? ( **Y** / **N** )  Date systemic treatment discontinued & tapering regimen: | | | | | | | | | | | | | | |
| Long term remission (off drops/systemic meds): ( **Y** / **N** ) | | | | | | | | | | | | | | |
| Required re-initiation of systemic medication ( **Y** / **N** ) | | | | | | | | | | | | | | |
| Recapture ( **Y** / **N** ) | | | | | | | | | | | | | | |
| **Disease Activity/year pre/post-biologic treatment initiation: ___________________**(date MM-DD-YYYY) | | | | | | | | | | | | | | |
| Year | Total Visits | | Visits w/active disease OD | | | | Visits w/active disease OS | | | | | | Time interval (dates) | |
| -2 |  | |  | | | |  | | | | | |  | |
| -1 |  | |  | | | |  | | | | | |  | |
| +1 |  | |  | | | |  | | | | | |  | |
| +2 |  | |  | | | |  | | | | | |  | |
| +3 |  | |  | | | |  | | | | | |  | |
| +4 |  | |  | | | |  | | | | | |  | |
| +5 |  | |  | | | |  | | | | | |  | |
| +6 |  | |  | | | |  | | | | | |  | |
| +7 |  | |  | | | |  | | | | | |  | |
| +8 |  | |  | | | |  | | | | | |  | |
| +9 |  | |  | | | |  | | | | | |  | |
| +10 |  | |  | | | |  | | | | | |  | |

**Visit Activity**

**-2 to -1 year pre-IFX**

| **Date** | **OD activity** | **OS activity** | **Assessment/Plan** |  |
| --- | --- | --- | --- | --- |
|  |  |  |  |  |
|  |  |  |  |  |
|  |  |  |  |  |
|  |  |  |  |  |
|  |  |  |  |  |
|  |  |  |  |  |
|  |  |  |  |  |
|  |  |  |  |  |
|  |  |  |  |  |
|  |  |  |  |  |

**-1 to 0 years pre-IFX**

| **Date** | **OD activity** | **OS activity** | **Assessment/Plan** |  |
| --- | --- | --- | --- | --- |
|  |  |  |  |  |
|  |  |  |  |  |
|  |  |  |  |  |
|  |  |  |  |  |
|  |  |  |  |  |
|  |  |  |  |  |
|  |  |  |  |  |
|  |  |  |  |  |
|  |  |  |  |  |
|  |  |  |  |  |

**Year 0 to 1 on IFX**

| **Date** | **OD activity** | **OS activity** | **Assessment/Plan** | **Adherence** |
| --- | --- | --- | --- | --- |
|  |  |  |  |  |
|  |  |  |  |  |
|  |  |  |  |  |
|  |  |  |  |  |
|  |  |  |  |  |
|  |  |  |  |  |
|  |  |  |  |  |
|  |  |  |  |  |
|  |  |  |  |  |
|  |  |  |  |  |

**Year 1 to 2 on IFX**

| **Date** | **OD activity** | **OS activity** | **Assessment/Plan** | **Adherence** |
| --- | --- | --- | --- | --- |
|  |  |  |  |  |
|  |  |  |  |  |
|  |  |  |  |  |
|  |  |  |  |  |
|  |  |  |  |  |
|  |  |  |  |  |
|  |  |  |  |  |
|  |  |  |  |  |
|  |  |  |  |  |
|  |  |  |  |  |

Year 2 to 3 on IFX

| **Date** | **OD activity** | **OS activity** | **Assessment/Plan** | **Adherence** |
| --- | --- | --- | --- | --- |
|  |  |  |  |  |
|  |  |  |  |  |
|  |  |  |  |  |
|  |  |  |  |  |
|  |  |  |  |  |
|  |  |  |  |  |
|  |  |  |  |  |
|  |  |  |  |  |
|  |  |  |  |  |
|  |  |  |  |  |

Year 3 to 4 on IFX

| **Date** | **OD activity** | **OS activity** | **Assessment/Plan** | **Adherence** |
| --- | --- | --- | --- | --- |
|  |  |  |  |  |
|  |  |  |  |  |
|  |  |  |  |  |
|  |  |  |  |  |
|  |  |  |  |  |
|  |  |  |  |  |
|  |  |  |  |  |
|  |  |  |  |  |
|  |  |  |  |  |
|  |  |  |  |  |

Year 4 to 5 on IFX

| **Date** | **OD activity** | **OS activity** | **Assessment/Plan** | **Adherence** |
| --- | --- | --- | --- | --- |
|  |  |  |  |  |
|  |  |  |  |  |
|  |  |  |  |  |
|  |  |  |  |  |
|  |  |  |  |  |
|  |  |  |  |  |
|  |  |  |  |  |
|  |  |  |  |  |
|  |  |  |  |  |
|  |  |  |  |  |

[This was repeated for additional years]
