# Supplementary material for: Effectiveness of long-term infliximab use and impact of treatment adherence on disease control in refractory, non-infectious pediatric uveitis
Source: Pediatr Rheumatol Online J. 2019 Nov 29;17:79. doi: 10.1186/s12969-019-0383-9 (PMC6884783; doi:10.1186/s12969-019-0383-9)
Supplement: Supplementary file 2 — Additional file 2: Table S2. Demographic, Clinical and Treatment History of Non-Responders (or Loss of Initial Response) to Infliximab Therapy. The demographic, clinical and treatment history of patients who failed IFX for arthritis or uveitis are listed. As stated in the text, no major trends were noted, however, a high proportion had associated systemic disease. JIA = Juvenile idiopathic arthritis; ANA + = anti-nuclear antibody positive; RF- = Rheumatoid factor negative; PA = polyarticular; OU = both eyes, mos = months; OD = right eye; OS = left eye; IFX = infliximab; PA = prednisolone acetate 1%; MTX = methotrexate; ADA = adalimumab; N/A = not applicable; HACAs = human antichimeric antibodies. [file 12969_2019_383_MOESM2_ESM.docx]

Additional file 2: Table S2. Demographic, Clinical and Treatment History of Non-Responders (or Loss of Initial Response) to IFX

| **Patient Demo-graphics** | **Systemic Association** | **Age of JIA Diagnosis**  **(mos)** | **Age of uveitis diagnosis (mos)** | **Duration of uveitis prior to IFX start**  **(mos)** | **Previous treatments** | **Uveitis location/laterality** | **Arthritis active pre/MRV exam?** | **Uveitis active pre/MRV exam?** | **Reason for Discontinuation** | **Treatments after IFX**  **(if known)** |
| --- | --- | --- | --- | --- | --- | --- | --- | --- | --- | --- |
| Male, African American | None | N/A | 150 | 12 | MTX | Pan-uveitis/ OU | N/A | Yes/Yes | -Uveitis control requiring > 2 drops of PA daily  + HACAs | No treatment, lost to follow-up |
| Female,  Caucasian | JIA, ANA+ RF neg PA | 18 | 21 | 25 | MTX | Anterior/  OU | Yes/Yes | Yes/Yes | -Uveitis control requiring > 2 drops of PA daily  -JIA not controlled  +HACAs | 1. ADA q2weeks, qweekly + MTX  2. Abatacept + Leflunamide  3. Actemra + Leflunamide |
| Male, Caucasian | JIA  (enthesitis, HLA-B27 positive) |  | 140 | 109 | ADA 40 mg q1week  MTX | Anterior/  OU | No/No | Yes/Yes | Active uveitis | Golimumab |
| Female, Caucasian | JIA (psoriatic, ANA+) | 21 | 45 | 94 | ADA 40 mg q2weeks | Anterior/OU | Yes/Yes | Yes/No | Uveitis  Arthritis  +HACAS | 1. Abatacept  2. Adalimumab 40 mg q1week |
| Female, Caucasian | JIA (ANA+, RF-, PA) | 30 | 33 | 124 | ADA 40 mg q1week  MTX | Anterior/OU | Yes/Yes | Yes/No | Active arthritis | Golimumab |
| Hispanic, Male | JIA (ANA+, RF-, PA) | 14 | 65 | 59 | ADA 40 mg q2 weeks  MTX | Anterior/OU | Yes/Yes | No/No | Active arthritis | Unknown |

JIA = Juvenile idiopathic arthritis; ANA + = anti-nuclear antibody positive; RF- = Rheumatoid factor negative; PA = polyarticular; OU = both eyes, mos = months; OD = right eye; OS = left eye; IFX = infliximab; PA = prednisolone acetate 1%; MTX = methotrexate; ADA = adalimumab; N/A = not applicable; HACAs = human antichimeric antibodies
