# Supplementary material for: Effectiveness of long-term infliximab use and impact of treatment adherence on disease control in refractory, non-infectious pediatric uveitis
Source: Pediatr Rheumatol Online J. 2019 Nov 29;17:79. doi: 10.1186/s12969-019-0383-9 (PMC6884783; doi:10.1186/s12969-019-0383-9)
Supplement: Supplementary file 3 — Additional file 3: Table S3. Ocular Complications Per Eye Pre and During Infliximab treatment (Per Eye, Right eye n = 25, Left eye n = 25). Ocular complications present before and while on IFX are listed by eye (as opposed to by patient) involved. We did statistical analysis on the complications per patient, please refer to Table 2. Abbreviations: OD – right eye, OS – left eye. *Cataracts that required surgery while on IFX were present prior to IFX initiation. In both patients, absolute control of inflammation off topical steroids prior to cataract surgery was rationale for the decision to start IFX. ** Glaucoma surgery was performed in one of these patients within a month of starting IFX. [file 12969_2019_383_MOESM3_ESM.docx]

Additional file 3: Table S3. Ocular Complications Per Eye Pre and During Infliximab treatment (Per Eye, Right eye n = 25, Left eye n= 25)

|  | Pre-IFX OD | During-IFX OD | Pre-IFX OS | During IFX OS |
| --- | --- | --- | --- | --- |
| Band keratopathy | 3 | 0 | 1 | 0 |
| Cystoid Macular Edema | 2 | 0 | 6 | 0 |
| New cataract diagnosis | 13 | 3 | 8 | 5 |
| New glaucoma diagnosis | 7 | 0 | 7 | 0 |
| New glaucoma suspect diagnosis | 10 | 0 | 11 | 0 |
| Cataract Surgery | 2 | 2* | 2 | 1* |
| Glaucoma Surgery | 5 | 2** | 3 | 1** |

Abbreviations: OD – right eye, OS – left eye

*Cataracts that required surgery while on IFX were present prior to IFX initiation. In both patients, absolute control of inflammation off topical steroids prior to cataract surgery was rationale for the decision to start IFX.

** Glaucoma surgery was performed in one of these patients within a month of starting IFX.
